# Addressing fragility through community-based health programmes: insights from two qualitative case study evaluations in South Sudan and Haiti

**DOI:** 10.1186/s12961-019-0420-7

**Published:** 2019-02-14

**Authors:** Séverine Erismann, Sibel Gürler, Verena Wieland, Helen Prytherch, Nino Künzli, Jürg Utzinger, Bernadette Peterhans

**Affiliations:** 1 Swiss Tropical and Public Health Institute, P.O. Box, CH-4002 Basel, Switzerland; 2 University of Basel, P.O. Box, CH-4003 Basel, Switzerland; 3 swisspeace, CH-3013 Bern, Switzerland; 4 Swiss Red Cross, CH-3011 Bern, Switzerland

**Keywords:** Fragile and conflict-affected states, fragility, Haiti, health programmes, South Sudan, stability, water, sanitation and hygiene (WASH)

## Abstract

**Background:**

Fragility can have a negative effect on health systems and people’s health, and poses considerable challenges for actors implementing health programmes. However, how such programmes, in turn, affect the overall fragility of a context is rarely considered. The Swiss Red Cross has been active in South Sudan and Haiti since 2008 and 2011, respectively, and commissioned a scoping study to shed new light on this issue within the frame of a learning process launched in 2015.

**Methods:**

The study consisted of a document review, qualitative field research undertaken between June and August 2015 in South Sudan and Haiti, and two data triangulation/validation workshops. Semi-structured key informant interviews and focus group discussions included 49 purposively sampled participants who helped build a deeper understanding of what constitutes and drives fragility in the respective countries. Moreover, interviews and focus group discussions served to grasp positive and negative effects that the Swiss Red Cross’s activities may have had on the overall state of fragility in the given contexts.

**Results:**

Qualitative data from the two case studies suggest that the community-based health programmes implemented in South Sudan and Haiti may have influenced certain drivers of fragility. While impacts cannot be measured or quantified in the absence of a baseline (the projects were not originally designed to mitigate overall fragility), the study nevertheless reveals entry points for designing programmes that are responsive to the overall fragility context and contain more specific elements for navigating a more sustainable pathway out of fragility. There are, however, multiple challenges, especially considering the complexity of fragile and conflict-affected contexts where a multitude of local and international actors with different goals and strategies interfere in a rapidly changing setting.

**Conclusions:**

Health programmes may not only reach their health objectives but might potentially also contribute towards mitigating overall fragility. However, considerable hurdles remain for aid agencies, especially where scope for action is limited for a single actor and where engagement with state structures is difficult. Thus, cooperation and exchange with other aid and development actors across the spectrum has to be strengthened to increase the coherence of aid policies and interventions of actors both within and across the different aid communities.

## Background

The provision of healthcare in fragile and conflict-affected states that are home to over two billion people in 2018 remains a significant concern not only to health sector actors but also to the broader state and peace-building community [1, 2]. While there is no universal definition of fragile contexts, most policy-makers and donors focus on poor state performance, i.e. weak state capacity as well as weak state legitimacy [3], which leaves their populations vulnerable to a wide range of threats and shocks, including extreme poverty and a lack of security and essential services. The 2007 OECD Development Assistance Committee Principles for Good International Engagement and the 2011 New Deal Principles developed to improve policies and practice of aid organisations call for interventions that are not only adapted to confront context-specific challenges but, more importantly, also strive to help building legitimate, resilient and effective state institutions to ensure a sustainable transition out of fragility [4, 5].

Health actors agree that fragile contexts pose important challenges in implementing health interventions and programmes. There are strong interlinkages between the dimensions of fragility (societal, political, economic, environmental and security) [6], health systems, and people’s health and well-being [2]. Health systems in fragile states are often characterised by an inability to provide basic health services, particularly in rural areas. Most common issues include a lack of necessary infrastructure, a dearth of skilled human resources, and inadequate capacity-building mechanisms to build robust health systems and ensure adequate coordination and oversight of health services by the government [7]. Generally, health programmes are informed mainly by technical approaches geared towards reaching defined health objectives in a given context. While health actors operating in fragile environments do consider the extreme challenges of working in fragility and try to find solutions to cope with absent or underperforming state actors (working around fragility) by adopting bottom-up approaches, they rarely question whether and to what extent community health programmes influence (positively or negatively) the overall fragility context and whether health programmes should, beyond health objectives, also incorporate strategies aimed at reducing overall fragility (i.e. working on fragility) [8, 9].

Thus far, the broader implications of such programmes and whether they link to rather top-down peace- and state-building efforts aimed at reducing fragility have not been systematically explored. Indeed, there is a lack of both research and evidence that could guide effective and community-based health interventions in such complex environments [7, 8, 10, 11].

There is continued interest of the donor community to invest in the health sector in fragile contexts [12–14]. Hence, there is a need to better understand how to design and implement effective health programmes in fragile contexts, to improve decision-making and to further the debate among both health and peace actors. This would allow for more coordinated approaches and harmonised aid policies aimed at sustainably mitigating fragility [15]. This is all the more relevant considering the abundance of national and international actors who, in rapidly changing situations, often work simultaneously on humanitarian relief, development and peacebuilding [16].

Against this background, the Swiss Red Cross (SRC) initiated a learning process in 2015 and commissioned two case study evaluations of community-based health programmes in South Sudan and Haiti. The objectives of the case studies were to better understand the key factors, dynamics and actors driving fragility, considering, beyond the health dimension, the socio-political and economic environment in the given contexts. This, in turn, would allow for further assessment of how the SRC’s interventions interacted with the broader fragility context to ultimately determine whether a sectoral health programme can – beyond reaching its health targets – contribute to mitigating fragility in the longer term.

### Context of the SRC case studies

**Fig. 1:**
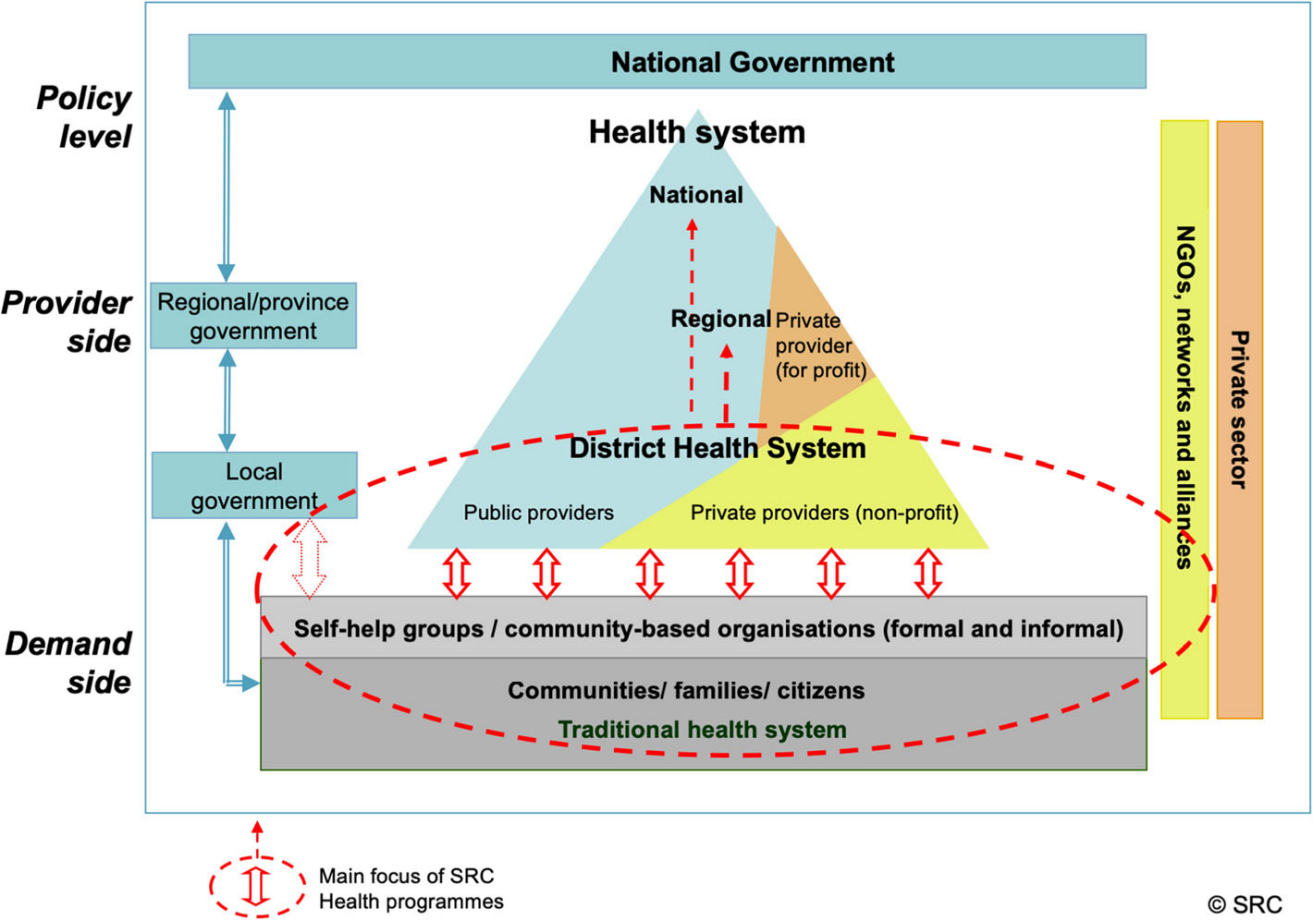
Swiss Red Cross’s (SRC) health programme approach

The SRC selected South Sudan and Haiti to serve as case studies as they are among the target countries of their long-term investments. Both countries are profoundly affected by myriad aspects of fragility. According to the classification of the Fund for Peace Fragility Index in 2017 [17], South Sudan is ranked as the most fragile state in the world, while Haiti is on position 11 among 178 listed countries. The SRC health programmes implemented in South Sudan and Haiti aimed at building bridges between service providers, national and local authorities, and communities (Fig. 1). The programmes pursued multiple approaches with an emphasis on enhancing quality and improving access to health services in South Sudan and improving water supply and safe sanitation in Haiti. In both countries, they sought to promote capacity-building at the individual, community and health systems level. The following sections give a brief overview of the two contexts and the SRC’s health programmes implemented.

### South Sudan

South Sudan is the world’s youngest nation, with an estimated population of 12 million in 2015 [18]. Following the 2005 Comprehensive Peace Agreement and after more than two decades of civil war in Sudan, South Sudan became a sovereign state in July 2011. Civil conflict resumed in December 2013 with heavy fighting starting from Juba before rapidly spreading to other parts of the country [19]. Since 2013, an additional 200,000 people were forced to flee, bringing the total displaced people to 2.4 million [20]. At the time of manuscript writing in the second half of 2018, peace talks between the government and the opposition were on-going, yet the situation in the country remains highly volatile [21]. South Sudan is one of the poorest countries in the world, with almost two-thirds of its population (65.9%) living below the poverty line (US$ 1.90) [22]. According to the Health Sector Development Plan from the Ministry of Health (MoH) in 2012, between 20% and 30% of the population had access to health services, 80% of which were provided by non-governmental organisations (NGOs). Child and maternal mortality rates were very high; estimates for the year 2006 of under-five mortality rate was 135 per 1000 live births, while maternal mortality rate was 2054 per 100,000 live births [23]. For comparison, the global estimate of under-five mortality was 61.6 per 1000 live births and that of maternal mortality rate was 277 per 100,000 in the same year of investigation [24, 25].

**Fig. 2:**
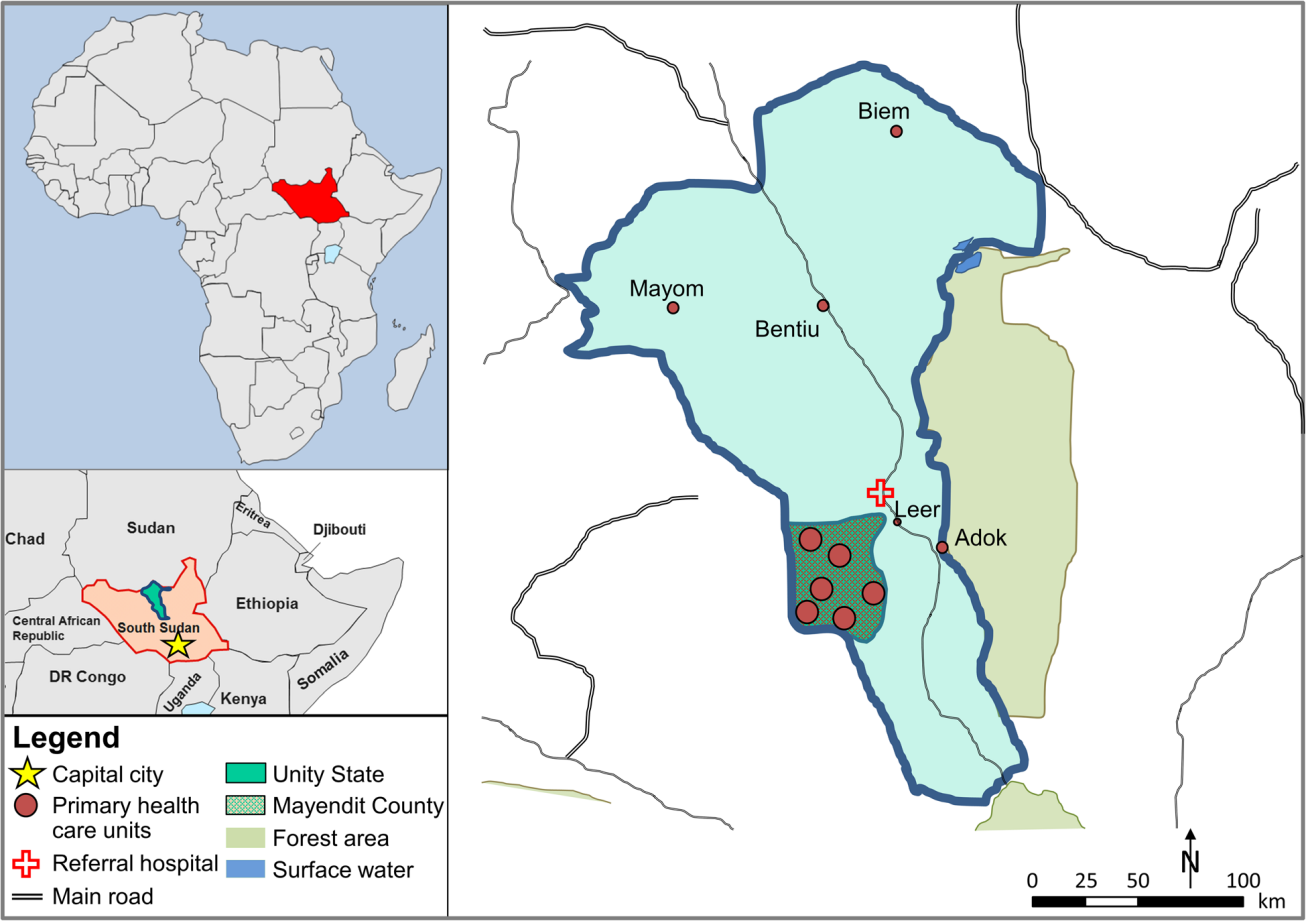
Study sites of the case study evaluation in South Sudan, July 2015

It is in this context that the SRC implemented a community-based healthcare project in Mayendit county (Unity state) (Fig. 2). According to an initial health and needs assessment conducted by the SRC prior to the onset of the project, Mayendit county had extensive health needs, with no primary healthcare units, one primary healthcare centre and one referral hospital in the neighbouring Leer county. The project’s aim was therefore to improve access to basic healthcare services of good quality in this highly underserved region, focusing on women of reproductive age, children and marginalised persons/groups. It was launched in 2008 as part of the SRC programme in the post-conflict situation in southern Sudan and was implemented post-independence from 2011 to 2013 with the newly established health authorities and the South Sudan Red Cross (SSRC) as its main counterpart. The SRC project focused on constructing and equipping primary healthcare services, providing training and capacity-building for health staff, and on the sustainability of financing and service provision. By November 2013, six primary healthcare units were established and equipped with essential infrastructure and staff. Ninety percent of the people in Mayendit county had access to services of relatively good quality. At the community level, approximately 250 SSRC volunteers and 27 village (*boma*) health committees were trained with various prevention activities supported (Table 1).

**Table 1:** Summary of the Swiss Red Cross project objectives and outputs in South Sudan and Haiti

Country (period)	Project objectives	Project outputs
South Sudan (2008–2013)	Construction and equipment of primary healthcare services, providing training and capacity-building for health staff, and on sustainability of financing and service provision through local authorities	• Construction and equipment of six primary healthcare units • Training and on-the-job coaching of MoH health staff in the units • Training of 250 SSRC volunteers • Training of 27 village (*boma*) health committees
Haiti(2011–2017)	Provision of clean water at the household and the community level, implementation of hygiene promotion activities and construction of latrines at household (WASH 1) and at community level (WASH 2)	• 10 hygiene and health promotion training sessions conducted for 123 members of the EIC • Construction of 1206 latrines • Construction of 508 rainwater catchment systems for households and two for schools • Repair of 160 damaged water reservoirs • Disinfection of 362 latrines

*EIC* ‘Equipes d’Intervention Communautaires’, *MoH* Ministry of Health, *SSRC* South Sudan Red Cross, *WASH* Water, sanitation and hygiene

Shortly before the project’s planned handover to the health authorities in late 2013, heavy fighting broke out and, hence, the project was stopped. Access to the project area was no longer granted. Consequently, the SRC adapted its programme to respond rapidly to the humanitarian needs, switching from its development approach to emergency aid, but maintaining the SSRC as its key implementing partner. At the same time, the SRC provided support to the International Federation of Red Cross and Red Crescent and the International Committee of the Red Cross emergency relief operations.

### Haiti

State fragility and the absence of a government providing services to the population had marked the history of Haiti long before a major earthquake occurred in 2010 [26]. Since its independence in 1804, the Republic of Haiti has faced a multitude of challenges, including having to repay France, the nation’s former colonial power, the equivalent of US$ 21 billion in today’s value in exchange for the loss of valuable plantations, property and slaves, and for the recognition of national sovereignty. It took Haiti until 1947 to pay for its freedom, using tax revenues [27]. Furthermore, Haiti has been the backdrop for oppressive government regimes (legacy of Duvalier’s family repressive dictatorships from 1957 to 1986) with little economic development, persistent human capital flight and widespread corruption [27, 28]. Due to growing concerns over corruption, the international community began to change its aid policy, providing direct aid through NGOs and bypassing the national authorities. Parallel structures were established by an ever-increasing number of aid organisations working in substitution of the government [29].

Haiti is not only affected by political instability, but the country is also highly vulnerable to recurrent natural disasters (e.g. tropical storms and floods), with over 90% of its population being at risk [30]. With a magnitude of 7.0, the earthquake that struck on 12 January 2010 was unprecedented in its severity. Over 3 million people were affected, of whom an estimated 220,000 people died and 1.3 million lost their homes [31]. The healthcare system was inadequate even before the disaster, yet after the earthquake, 37 of Haiti’s 48 hospitals were forced to discontinue their services [32]. Moreover, a cholera outbreak spread rapidly across the country, with 8534 deaths reported by the Haitian Ministry of Public Health and Population [33]. As of 2016, 59% of Haiti’s population, estimated at 10.4 million inhabitants, lived below the national poverty line of US$ 2.4, and the country still depended mostly on foreign financial and technical support [30]. Its coverage level in the areas of safe water supply and sanitation are the lowest in the western hemisphere, with 36% of Haitians lacking access to clean water and 69% to essential sanitation services [30].

**Fig. 3:**
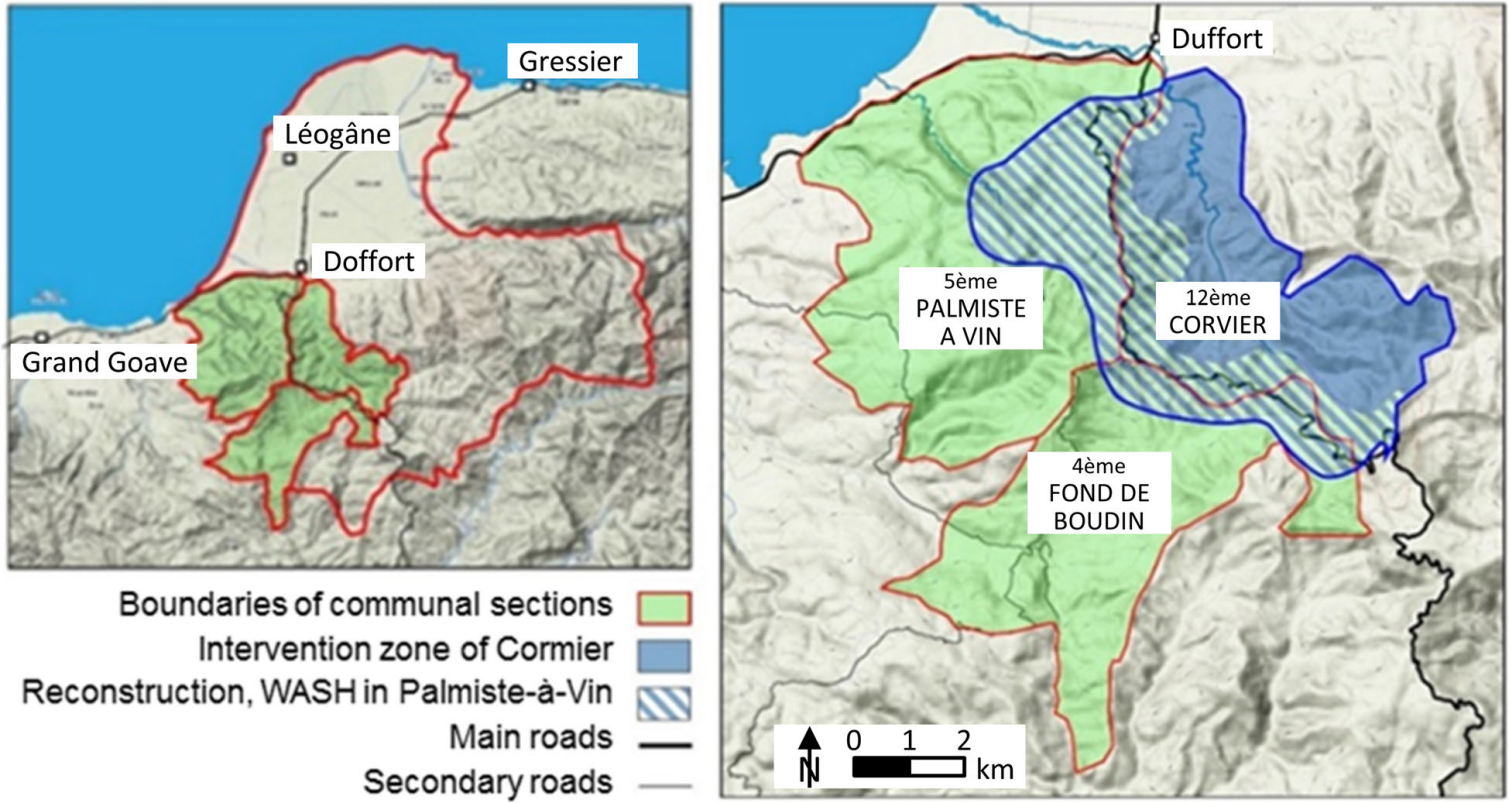
Study sites of the case study evaluation in Haiti, July 2015

To address these challenges, the SRC started working in Haiti in the aftermath of the 2010 earthquake. During the emergency phase, SRC’s engagement was centred on basic health services to improve mother and child care and cholera control. Subsequently, the SRC started to lay the groundwork for the reconstruction phase with the building of semi-permanent shelters in the ‘Section Communale Palmiste-à-Vin’ in the Léogâne district. As part of the housing reconstruction programme, a community-based water, sanitation and hygiene (WASH) project was launched in 2011. A second WASH project was implemented from 2014 onwards in the neighbouring ‘Section Communale de Cormier’ as part of the long-term development programme (Fig. 3). The main components of the two WASH projects consisted of the construction of latrines at household (WASH 1) and community level (WASH 2). The provision of clean water at the household (rainwater catchment) and the community level (water cisterns), as well as hygiene promotion activities for the prevention of cholera and other water-borne diseases, were implemented as part of both project components. Project beneficiaries contributed workforce for the construction of latrines and water catchment systems. The SRC’s main partners were the Haiti Red Cross Society and the National Water and Sanitation authorities (Direction Nationale de l’Eau et de l’Assainissement) under the Ministry of Public Works. Hygiene promotion activities were implemented by the ‘Équipes d’Intervention Communautaires’ (EIC), a group of village volunteers created as the most decentralised level of disaster response by Haiti’s Civil Protection Authorities (‘Direction de la Protection Civile’; DPC) and the Haiti Red Cross in cooperation with various international aid agencies in different areas of Haiti. In 2013, declaring the end of the emergency phase, the Ministry of Public Works issued a new policy stipulating that, by the end of 2014, the construction of household latrines should no longer be subsidised by aid agencies [34]. The SRC integrated this new policy into WASH 2, ending the free construction of latrines at the household level. The SRC also decided to discontinue the provision of free construction material for household water catchment systems and the distribution of free hygiene promotion materials in order to transition from reconstruction to a more developmental outlook with beneficiaries and communities taking on more responsibilities and ownership of project activities in their area. Together, the two WASH projects conducted 10 hygiene and health promotion training sessions for 123 members of the EIC tasked with raising awareness among the local communities, constructed 1206 latrines and 508 household rainwater catchment systems (two for schools), repaired 160 damaged water reservoirs, and disinfected 362 existing latrines (Table 1).

## Methodology

### Study design and definitions

A qualitative study design, which incorporated field research, the conduct of interviews and focus group discussions (FGDs) as well as data triangulation/validation workshops, was chosen to examine the two SRC case studies in two different contexts. The specific projects under study were (1) the community-based healthcare project in Mayendit county, South Sudan (implemented from 2008 to 2013), and (2) the health and hygiene promotion activities (WASH 1 and 2) in Léogâne, Haiti (WASH 1 implemented from 2011 to 2014 and WASH 2 initiated in 2014 and ongoing during the case study evaluation). While the projects were designed to be implemented in fragile contexts, i.e. having to cope with weak structures and effects of crisis, they were not explicitly designed to tackle and mitigate root causes or drivers of fragility in their specific context.

The aims of the case studies were (1) to deepen the SRC’s understanding of fragility by identifying key factors, dynamics and actors driving overall fragility in the two case study contexts, and (2) to assess the interactions between the health programmes and the fragility context in each case study context to gauge the SRC’s scope of working on fragility, and to provide input for a more holistic SRC health strategy and policy.

Definitions of what constitutes fragility and conflict-affected states vary in the literature and among different stakeholders [1, 8, 14, 35]. The starting point for the further refinement of the SRC’s understanding of ‘fragility’ as well as for the analysis and interpretation of the findings was the SRC’s definition of fragile states and fragile contexts, which were based on the definitions used by the Swiss Agency for Development and Cooperation (SDC) and the OECD Development Assistance Committee (Table 2).

**Table 2:** Swiss Red Cross definitions of fragility and conflict-affected states in 2015

Fragile context/situation	Describes a context, which is characterised by weak or unstable institutions, poverty, violence, corruption and political arbitrariness (adapted from SDC’s “Characteristics of fragile contexts” [3])
Fragile state	Has a weak capacity to carry out basic functions of governing a population and its territory, and lacks the ability to develop mutually constructive and reinforcing relations with society. As a consequence, trust and mutual obligations between the state and its citizens have become weak (based on definition from OECD/DAC 2011) [72]

*DAC* Development Assistance Committee, *OECD* Organisation for Economic Co-operation and Development, *SDC* Swiss Agency for Development and Cooperation

The original data for the scoping study were collected between June and August 2015 in South Sudan and Haiti by external experts from the Swiss Tropical and Public Health Institute, swisspeace and an SRC health expert. The data were supplemented with information from the SRC’s project documents and the extant literature, as summarised in Table 3.

**Table 3:** Overview of data collection methods by country

Data source	Country	
	South Sudan	Haiti
Key informant interviews (n)	Yes (19)	Yes (14)
Focus group discussions (n)	No	Yes (4, total of 16 participants)
Data triangulation/validation workshops	Yes (held at SRC headquarters in Bern, Switzerland with SRC, SSRC and SDC staff)	Yes (held in Haiti with entire SRC team across various departments)
Document review	Yes	Yes

*SDC* Swiss Agency for Development and Cooperation, *SRC* Swiss Red Cross, *SSRC* South Sudan Red Cross

### Sampling, data collection methods and analysis

Interview and FGD participants were initially identified and purposively selected with the help of the SRC from among the beneficiaries, SRC staff and implementing partners of the SRC health programmes. Additionally, whenever possible, authorities from the local and national levels and other stakeholders working in the two case study contexts were invited to participate. However, due to violent outbreaks and on-going conflict in South Sudan, the evaluators did not have access to either the SRC project areas in Mayendit county or the project beneficiaries. Thus, instead, key informant interviews with project implementers and authorities were conducted in the capital Juba.

Overall, the two case studies included 49 participants (13 females and 36 males; 19 from South Sudan and 30 from Haiti); participant affiliations are summarised in Table 4. Of note, most of the participants were part of the SSRC, the SRC or a partner organisation, while only few beneficiaries were interviewed (exclusively in Haiti).

**Table 4:** Stakeholders interviewed during the fact-finding mission in South Sudan and Haiti, 2015

Affiliation	Type of interview	Nb. of participants
South Sudan		
SSRC headquarters Juba	Interview	2
SSRC Bentiu branch	Interview	2
SSRC Juba	Interview	3
IFRC	Interview	1
ICRC	Interview	3
SRC	Interview	2
SDC	Interview	4
MSF	Interview	1
Unity State Ministry of Health	Interview	1
Haiti		
HRC	Interview	2
PFST	Interview	1
SRC Haiti	Interview	8
SRC headquarters (Bern)	Interview	1
EICs	FGD	6
WASH 1 and WASH 2 beneficiaries	FGD	5
Local authorities (Ministry of Internal Affairs)	Interview	2
Health authorities (MSPP)	Interview	1
WASH authorities (Ministry of Public Works)	Interview	1
IFRC (Haiti)	Interview	2
ICRC (Haiti)		
SDC (Haiti)	Interview	1

*EIC* Équipes d’Intervention Communautaires, *FGD* focus group discussion, *HRC* Haitian Red Cross, *ICRC* International Committee of the Red Cross, *IFRC* International Federation of Red Cross and Red Crescent, *MSF* Médecins Sans Frontières, *MSPP* Ministère de la Santé Publique et de la Population, *PFST* Congrégation des Petits Frères de Saint Thérèse, *SDC* Swiss Agency for Development and Cooperation, *SRC* Swiss Red Cross, *SSRC* South Sudan Red Cross, *WASH* water, sanitation and hygiene

A semi-structured interview guide was developed with open-ended questions to allow for incorporation of new topics during the interviews. The guide defined the following areas to be explored with the participants: (1) analysis of the overall fragility context (socio-political and economic dimensions) to improve the understanding of context-specific aspects of fragility (key issues and dynamics driving fragility and actors involved); (2) assessment of the interactions between the health programme and the overall fragility context by exploring how the health programme was affected by the broader fragility context and vice versa; and (3) staying engaged – what are possible entry points for a long-term engagement that may promote a transition out of fragility?

The logic and structure of the interview guide was modelled after the ‘3-Step Approach for Working in Fragile and Conflict-Affected Situations’, developed by HELVETAS Swiss Intercooperation and swisspeace [36]. The 3-Step Approach was developed in 2013 as a framework for policy-makers and aid practitioners to promote a deeper understanding of the context in which they are operating to allow for improved policy and practice. A number of aid actors, including Swiss NGOs, SDC, and United Nations agencies have since adopted the model. The interview guide was pre-tested with context-experienced persons during a preparatory workshop together with the SRC before the fieldwork.

Key-informant interviews and FGDs were conducted by the external evaluator in South Sudan and by an external evaluator together with an SRC health expert for Haiti. Each interview and FGD lasted 60–120 min. Interviews were held in English (South Sudan) and French or Creole (Haiti). In Haiti, interviews and FGDs were done face-to-face, while for the South Sudan case study participants were also approached by either telephone or email.

Two workshops were conducted to triangulate and validate data collected for each of the case studies; this was especially important for data collected in South Sudan, where the fieldwork was hampered by the lack of access to both project location and beneficiaries. The South Sudan workshop was held in July 2015 in Bern, Switzerland, with members of the SRC, SSRC and SDC shortly after original data had been collected in Juba. With regard to Haiti, a 4-day workshop was conducted with the entire SRC staff in Haiti (across various departments) during the field research.

The interviews were transcribed by the evaluators using Microsoft Word. Thematic content analysis was performed (based on a qualitative case study methodology) [37]. Together with the outputs from the data triangulation and validation workshops, the themes identified were grouped into the concepts and categories to be further translated into the findings. Reporting adheres to COREQ criteria for qualitative research [38].

Project evaluation reports and strategic documents (South Sudan: health need assessment report 2007, mid-term review 2009, phase-out strategy 2012; Haiti: final project reports 2011 to 2014; project document WASH 2014 to 2017 and country programme strategy 2013–2017) as well as relevant literature were also examined for data triangulation. The output document of the overall SRC learning process and the case study evaluations is a paper entitled ‘Think differently and stay engaged: health programming in fragile contexts’, which served as a reference for the development of this manuscript [39].

## Results

### Conceptualising and contextualising fragility

Based on the interviews conducted, primary drivers of fragility identified for both settings were (1) inability or unwillingness of the state to provide basic services; (2) lack of effective mechanisms to ensure inclusive citizen participation; (3) erosion of social cohesion and community spirit; and (4) high external aid dependency. The identified key drivers are explained and contextualised in Table 5.

**Table 5:** The broader context of fragility in the two case studies

Identified key drivers	Literature	Context Haiti	Context South Sudan
Inability or unwillingness of the state to provide basic services	Failure of a state to establish itself as a service provider (e.g. health services, physical security and economic development) drives fragility by poor overall governance and administration and are characterised by a lack of state representation at local levels and weak state-society relations [7, 57]	Study participants felt that the government has only weak roots at the local level and that much-needed services are mainly provided, if at all, by international actors. State–society relations are considered extremely weak. The government lacks legitimacy among a large part of the population and there is no real sense of citizenship	Study participants perceived a lack of the government legitimacy by civil society. The failure of state institutions to provide services in South Sudan is well documented, mainly attributed to the nascence of the state institutions, the inadequacy of fiscal transfers to lower tiers of government, and the lack of capacity among South Sudan’s public servants, institutions and organisations [58]
Lack of effective mechanisms to ensure inclusive citizen participation	States that lack effective mechanisms to ensure inclusive participation in the social, economic and political processes may be unable to meet social expectations of equitable distribution of and access to services and to manage social disruption, unrest or violence that may arise as a consequence [41]	The various stakeholders met in Haiti underlined the gap between the population and the political elite. Many feel excluded from any decision-making process. Moreover, the country is plagued by corruption at all levels. Control mechanisms are lacking to prevent personal enrichment by the elite (“*10% have everything and 90% have nothing*”). Rampant discontent is evidenced by recurring and sometimes violent manifestations and strikes	The stakeholders interviewed felt that public satisfaction and citizen participation were enhanced at county level through the implementation of needs-based and locally accepted and adapted programme strategies. However, in South Sudan, since its independence in 2011, institutional mechanisms have been insufficiently in place to foster trust and civil society inclusion [59]. Perceptions are widespread of government’s malfeasance, self-interest and disregard for citizen priorities [59]. Violent tribal clashes in the project area occurred frequently
Erosion of social cohesion and community spirit	Social cohesion refers to the capacity of a society to ensure the welfare of its members, minimising disparities and avoiding polarisation. Social cohesion is often considered as a protective factor that confers some resilience upon communities [60, 61]. The absence of social cohesion in society contributes to overall insecurity, lack of trust between groups and may prevent states from establishing a robust governing system, contributing, in turn, to the fragility of state institutions [62]	According to the study participants met in Haiti, it has become difficult to encounter community spirit among the population. Traditional systems of mutual help and support, such as rural community work (*Konbit*), have been displaced by cash-for-work projects implemented by international aid agencies since long before a major earthquake in 2010. A World Bank report from 2006, moreover, stated that population shifts from rural to urban areas place a high burden on state institutions to provide basic services in the face of a loss of social cohesion [63]. After the 2010 earthquake, interpersonal trust decreased even further due to the vulnerabilities of the displaced [64]	Since before its independence, the southern part of Sudan has been war-torn for several decades, with a number of war-disabled persons, broken-up families, eroded cultural patterns and social cohesion and losses of assets [65]. Study participants in South Sudan felt that, even though the project had no explicit strategy to tackle the loss of social cohesion through decades of violent conflict, it contributed to building trust among various local stakeholders through its activities
High external aid dependency and weak coordination	Long-term humanitarian relief assistance and development aid fail to promote efficient government institutions and sustainable economic development, especially when other forms of international engagement with crisis are absent that would address root causes or when the capacity of states to absorb and equitably manage large resource flows is reduced [66–68]	The various participants felt that Haiti’s chronic dependence of external aid is largely a result of long-term humanitarian relief assistance as well as the absence of economic reforms and international economic agreements. Furthermore, externally imposed standard structural adjustment programmes have harmed Haiti’s economy in the long run, according to many scholars [63]. Aid is, moreover, not systematically integrated into national budgets and structures, partly due to a lack of absorption capacities, and thus implemented through parallel structures, posing a real threat to sustainability [69]	According to study participants, a high aid dependency is largely due to the long-term international humanitarian engagement during the many years of civil war, often exacerbated by natural disasters. The aid system is fragmented, marked by weak coordination among partner organisations. During the Comprehensive Peace Agreement period and after independence in 2011, billions of dollars of aid and technical assistance to ‘build capacity’ in the nascent Government of South Sudan were provided by foreign development agencies [70]. Aid dependency, especially in the areas of food aid and health service delivery, is often considered as harming self-reliance and long-term development prospects [70, 71]

### Interaction of the SRC’s health programmes with the fragility contexts and potential contribution to addressing key factors driving fragility

#### Inability or unwillingness of the state to provide basic services

In general, respondents in South Sudan reported that health services were still primarily delivered by international NGOs and that, as a result, the government was found to have little legitimacy in the eyes of civil society. Several stakeholders noted, however, that the SRC, as other international NGOs, had started to shift their approach from direct project implementation to cooperation with state and local structures involved in health service delivery (MoH, local authorities and communities), which was considered an important step in the right direction for ensuring sustainability. The project implemented in Mayendit county succeeded in providing the authorities with an opportunity to assume a more active and responsible role and provided community members with a platform for regular meetings and direct engagement with government officials, albeit mainly from the local and regional levels. Nevertheless, several stakeholders highlighted that turnover is high among the MoH, leading to frequent changes in approaches and atmosphere. One interviewee said*:* “*The MoH supported the project with staff and technical inputs. However, the frequent change of staff, especially with a long-term project or when we were planning the handing over phase was highly problematic*”. Eventually, a solution was found between the MoH and the administrative and traditional authorities to improve cooperation. Overall, the SRC’s approach contributed to mitigating the effects of conflict on the community as health services continued to be delivered when political instability rose. In December 2013, the change in conflict dynamics, marked by the high levels of violence and mass displacement, resulted, nonetheless, in the sudden end of activities.

In Haiti, the absence of performing state institutions has been very noticeable throughout project implementation and engagement with government officials proved rather difficult. The SRC has been working mainly with local government structures, such as the ‘Conseil d’Administration de la Section Communale’ (CASEC) which, as the lowest level government representative (executive branch), is responsible for administering habitations at the local level. The SRC office has maintained some contacts at the central government level but not to the same extent as at the local level. Other than the CASEC, the SRC also maintains contact with the mayor’s office in Léogâne or the field technicians of the Ministry of Public Works, the ‘Techniciens en Eau Potable et en Assainissement pour les Communes’ for water catchment projects. As far as hygiene promotion activities are concerned the ‘Unités Communales de Santé’ (UCS) under the MoH would be another relevant counterpart for the SRC. However, the UCS in Léogâne has not yet developed any activities of its own, and the relationship between the UCS and the SRC remains at the level of a monthly coordination meeting where the SRC updates the UCS on its activities. The SRC, as one of the few organisations remaining in Léogâne, has, since the start of its programme, paid specific attention to the sustainability of their programmes beyond their presence. Trying to avoid creating too many parallel structures and with an exit strategy in mind, the SRC decided to make use of the EICs, which are the most decentralised level of disaster response of Haiti’s DPC and the Haitian Red Cross. This solution did not come without its challenges, as the linkages between the national level (DPC or Haitian Red Cross) and local level (EICs) have remained rather weak, affecting the sustainability aspect of the SRC’s exit strategy. During interviews and FGDs, the members of the various EICs of the Cormier area (WASH 2 programme) expressed their fear of not receiving the continued support of government agencies to carry out their activities once the SRC left. Overall, while the SRC involved state institutions in project implementation, engagement remained mainly at the local level and did not explicitly include elements that helped strengthen linkages between the local and national levels.

#### Lack of effective mechanisms to ensure inclusive citizen participation

Several respondents highlighted that the SRC health programme in South Sudan succeeded in implementing needs-based and locally adapted and accepted strategies, empowering the local population and fostering project ownership. Through the organisation of regular meetings with representatives of the different target groups, including local communities, traditional authorities, local and regional decision-makers, relationships of trust were built. By providing the project beneficiaries (i.e. members and stakeholders of the communities) the opportunity and experience to be included in an inclusive approach with real decision-making power, this could also be considered as a pathway towards citizen participation and rights, as many people do not even fully understand their rights. Public satisfaction and the concept of citizen participation were enhanced, at least at county level, as one interviewee noted: “*The community-based approach empowered the population on their health rights that increased demand and utilisation of healthcare and hence strengthened health services and equity in the project area*”.

As regards the Haiti case study, programme activities also attached great value to beneficiary participation and bringing together various stakeholders at the local level as a way of increasing ownership. In this sense, it could be said that the projects also helped achieve promotion of the concept of citizen participation, by providing the beneficiaries with a platform where concerns and ideas can be voiced and shared with the local authorities, albeit only at the local level. Some participants, however, stated that a wider range of local authorities should have been included in the project. While CASECs (executive branch of government) are involved and kept informed about the WASH project implementation, the Assemblée de la Section Communale, who officially act as elected representatives of their communities (comparable to a parliamentary structure) “*are not systematically involved by either SRC or other the international aid actors. When the international organisations arrived after the earthquake, they did not really know the Haitian context, the institutions and their roles. The NGOs should first contact the ASEC* [Assemblée de la Section Communale] *to get an overview of the area and the communities because it is the ASEC who live amongst them and know about their various needs. They know the areas much better than the CASEC*” (FGD EIC).

More generally about the inclusion of beneficiaries in decision-making processes within the context of aid programme implementation in Haiti, respondents expressed that projects proposed by international actors were rather guided by their organisations’ specialisations and competencies than the priorities of the communities. The fact that beneficiaries and authorities agreed to have WASH programmes implemented in their communities stemmed, for instance, more from a “*we will take what we get*” attitude and was not necessarily a priority according to the stakeholders interviewed. EIC team leaders involved in the implementation of the WASH 1 and 2 programmes, moreover, stated during a FGD that they would welcome a platform or mechanisms through which community representatives or local authorities could propose or suggest activities to international aid actors that would make sense for the communities.

#### Erosion of social cohesion and community spirit

While the project in South Sudan had no inbuilt and explicit strategies to tackle the loss of social cohesion through decades of violent conflict, it nonetheless contributed to building trust among various local stakeholders through its activities. Four participants from South Sudan mentioned that as the SRC and SSRC showed sensitivity to ethnic consideration (staff employment and deployment practices and through capacity-building with volunteers) which helped to reconnect people: “*The volunteers helped to build up trust in a fragmented civil society due to tribal conflicts and weak governance* […]”. This may indicate that, by providing services closer to the communities through participatory approaches, building relations of trust and showing sensitivity to ethnic considerations, social cohesion was strengthened throughout the SRC’s programme implementation.

The different stakeholders interviewed in Léogâne underlined that the spirit of self-help and mutual support had continually decreased with the arrival of international aid organisations already since the 1970s, especially with the introduction of cash-for-work projects. Social cohesion, moreover, further decreased after the 2010 earthquake following the displacement of a large number of people and in light of a massive increase of vulnerable groups and individuals among Haiti’s population. Changes in attitude were also noticeable during the implementation of the WASH projects in Léogâne according to the members of the EIC teams involved in the implementation of the activities. Particularly during WASH 2, which, marking a transition from relief to a more developmental assistance approach, no longer subsidised the construction of household latrines and discontinued the free distribution of hygiene promotion items, EICs were regularly confronted by the beneficiaries: “*[…] When we do door-to-door hygiene promotion activities, we are challenged by the beneficiaries about the fact that we are not distributing hygiene promotion items anymore (buckets, soaps and jerry cans.). They ask us, what is the SRC doing now? This is a big problem*” (FGD EIC). Rather harsh reactions of WASH 2 beneficiaries reflected their much more hard-set expectations and frustrations of not receiving the same level of assistance as previous WASH 1 beneficiaries in the neighbouring areas. The SRC’s WASH team in Léogâne stated that there is strong resistance among the population towards doing ‘unpaid’ work even if the own community or vulnerable members should benefit in the long term. This impression was also confirmed during the FGDs by the beneficiaries, the local authorities and the EIC teams involved in the hygiene promotion activities. Most interviewees said that discontent and tensions over perceived inequalities of aid distribution were a significant contributing factor to frustrations and tensions among and across communities. The failure and inability of the Haitian authorities to play a leading coordination role and to develop standardised approaches for aid programme implementation and inadequate coordination mechanisms among the various aid organisations constituted a great challenge also for the SRC. According to the FGD with WASH 1 beneficiaries, “*these different standards (of the various aid organisations) have created a lot of dissatisfaction and tensions among the beneficiary communities in the area*”.

#### High external aid dependency and weak coordination

As regards SRC health programmes’ potential contributions to decreasing external aid dependency in South Sudan, it needs to be recalled that the context is highly dependent on humanitarian aid as a result of long-term international humanitarian engagement. During the many years of civil war, often exacerbated by natural disasters, people lost their livelihoods and had to live in camps or move from place to place; this has gone on for so long that dependency rates are extremely high. Within the project area, the communities and authorities were often slow to react, as they were used to waiting for others to act rather than taking initiative themselves. Similarly, it was difficult to come up with a common vision for health service provision, as people were more focused on surviving for another day and unused to the concept of long-term planning. Service development depended mostly on foreign aid and on different stakeholders over a long period of time, making it difficult to achieve a coherent approach to health service delivery and impossible to avoid service fragmentation among the various aid actors. As one interviewee noted: “*There is an unequal relationship between the Red Cross movement partners. Although initiatives are needs driven, sometimes there is mistrust and confusion about who has what resources for what needs and who collaborates with whom.* […] *The imbalance in capacities to cope with the pressure of changing needs in fragile contexts can result in failed partnerships and weak coordination*”.

Several stakeholders in Haiti noted that long-term relief and development aid and the absence of economic reforms and international economic agreements that would benefit the entire population (instead of just the corrupt elite) had turned dependence on international aid into a stark reality. A growing disillusion has become apparent among a large part of the population who have increasingly adopted a ‘wait-and-see’ mentality, waiting for handouts. As one respondent highlighted: “[…] *We have gotten used that things will happen for us, arrive for us. The first important point would be that we become actors, responsible for our own development*”. Challenges to reverse aid dependency are, however, huge and individual actors such as the SRC have only little scope for action. Thus, the focus of the SRC programmes in Haiti has, in practice, thus far rather been on strengthening community resilience. Overall, with the sudden drop of aid agencies in Haiti and the lack of proper exit strategies and handover in respect of beneficiaries, local organisations or government counterparts, participants stated that there was a growing concern among the communities: “*Beneficiaries are looking to the future with concern, as there are no jobs or livelihood strategies that guarantee them sufficient income after the aid or development programme cease*” (FGD WASH1).

## Discussion

While the relationship between sectoral programmes and their linkages and possible contributions to state- and peacebuilding are under-researched, there is, unsurprisingly, also a lack of evidence on the potential impacts of health programmes on stability in fragile contexts [7, 8, 11]. Qualitative data from the two case studies presented here suggest that the community-based health programmes implemented in South Sudan and Haiti may have influenced certain drivers of fragility. While impacts cannot be measured or quantified in the absence of a baseline (the projects were not originally designed to mitigate overall fragility), the study nevertheless reveals potential entry points for designing programmes that are more considerate of the overall fragility context and that contain elements to help promote a more sustainable pathway out of fragility. However, challenges are abundant, especially considering the complexity of fragile and conflict-affected contexts where myriad local and international actors with different goals and strategies interfere in a rapidly changing setting. The findings of the case studies and implications are discussed, taking into consideration idiosyncrasies between South Sudan and Haiti regarding the underlying crisis.

With regards to the potential of strengthening the state as a service provider through sectoral health programmes, the findings from both case studies suggest that, while difficult, it is possible and necessary to integrate specific elements in the programme design that help promote state ownership and responsibility to safeguard sustainability in the longer run. Against the backdrop of weak government structures and institutions, apparent both in South Sudan and Haiti, the SRC engagement has focused on community-based health programmes as a means to strengthening community resilience and government institutions from the local (district) up to the national level and thus promoting their role as a service provider (Fig. 1). This has come with challenges in both contexts where state institutions are dysfunctional and only weakly rooted in the local levels. In the case of South Sudan, the strategy to strengthen community resilience, while engaging with state structures across all levels, looked promising. Nevertheless, after the handover of the project to SSRC and other local stakeholders, including the MoH, a sudden outbreak of violence in December 2013 resulted in the end of activities in Mayendit county. As for Haiti, already dysfunctional state structures and institutions were further incapacitated during the 2010 earthquake, where many state officials died and government buildings collapsed. Overall, it is not surprising that it is the international organisations that provide many services, albeit with adverse effects on state–society relationships. It is thus vital to work not only on building institutional capacity but also on the relationship between the state and the public.

In the frame of the current debate on fragility and how best to operate in fragile contexts [9, 10], the issue of whether to explicitly engage with state-building goals when implementing sectoral aid programmes raises some intriguing questions. From a development perspective, it may be argued that strengthening community resilience as an adaptive measure to adversity – and in light of a dysfunctional government – is necessary to achieve urgently needed health outcomes. Resilient actors (e.g. individuals and community networks) can draw on social, economic and environmental capital to adapt successfully and are thus able to moderate or avoid the negative consequences (develop coping strategies) of similar threats or to facilitate recovery after a traumatic event or disaster [40]. In terms of overall fragility, however, it is equally important to consider whether the continued absence of state-led development and services inhibits improvements in, or worsens, already weak state–society relations, where the core issues are trust and legitimacy [41]. This issue was discussed in a recent study conducted in Nepal, which found that strengthening communities can have adverse effects on state legitimacy (deep-rooted distrust towards state actors) [42]. However, the findings from the case study in South Sudan suggest that, by including government actors firmly into the design of a community-based programme and by providing quality health services, relationships with government authorities may be improved. However, previous studies underline that the quality of the service and equal access are key for enhancing state legitimacy [43–45].

As concerns the lack of effective mechanisms to ensure inclusive participation and foster social cohesion in both settings, the two case studies suggest that, when working in a participatory approach with different stakeholders, including government authorities, mutual trust and dialogue may be improved. While it is difficult for organisations such as the SRC to get involved in political reform, the idea and concept of citizen participation can be strengthened vis-à-vis the various groups involved and put into practice. Another important aspect that emerged in the Haiti case study is that there is a risk that the type of programme implemented in an area is determined rather by the range of services an aid organisation has to offer than by the local needs and priorities. Ideally, there should be mechanisms that enable communities to propose their own specific projects and solutions to be considered by the aid organisations and authorities (demand driven instead of supply driven).

Moreover, without coordination and agreement among government and aid actors on types and standards of interventions across the entire country, the opposite may be achieved and social cohesion may be undermined by fuelling tensions across the different beneficiary communities. It is thus vital to conceive projects with a good understanding of other aid actors, including their approaches and quality standards and to help push for coordination with other stakeholders by increasing advocacy. These findings are in line with other studies and reports on the humanitarian response in Haiti [15, 31, 46, 47]. Advocacy for better coordination and collaboration needs thus to be increased.

Moreover, it is important to recognise the controversy of attempting to foster social cohesion as part of a development programme, especially when implemented by external actors. Since building social cohesion relies on endogenous processes of building trust and inter-group relations, external actors need to clarify roles and responsibilities with the local stakeholders in a timely and culturally sensitive manner [48].

Entry points to help decrease overall dependency on external aid of fragile states are challenging to find for individual NGOs, which often do not have much leverage. To reduce root causes of aid dependency, other forms of engagement are warranted that include international actors from both public and private sectors, and which tackle the issue of the broader asymmetries of political and economic power in the global economic systems. Nonetheless, the scoping study found that, at the level of aid organisations, some measures should be taken to at least avoid further deterioration of the situation. Health programmes should be designed to be firmly anchored in, as far as possible, already existing and established state or community structures to ensure sustained local health service provisions. Developing intimate knowledge about the various actors present in a specific context may allow for new partnerships with less ‘conventional’ actors. For instance, it was suggested by SRC staff in Haiti that the *médecins feuille* could have been an interesting alternative to explore as a partner for the WASH programmes as they are the go-to person for every Haitian who has health worries. The *médecins feuille* are also represented at the central government level, albeit only recently. One issue that needs further scientific inquiry is the ‘Build Back Better’ approach, which has become a standard in reconstruction. As seen in the case of Haiti, it is important to note that a vast majority of material used during WASH programmes was bought in Haiti, but was ultimately imported. This is also true for material and goods that were used by other aid organisations that have been active in Haiti for several years. While it is true that local markets could not cope with the increased demand of goods during the influx of hundreds of aid actors, especially during the reconstruction phase, and did not meet international quality standards, some alternative avenues could nevertheless be further explored. For instance, for the construction of latrines, the SRC used, at least partially, high-standard components towards which the local market or the families could not contribute. It should therefore be carefully evaluated whether such high-quality standards as adopted by many, including the SRC, are truly necessary or whether there is some scope for using local materials and technologies that will allow communities and authorities to sustain and replicate activities more easily. Effective coordination and not bypassing but actively involving and strengthening local counterparts are particularly crucial for staying engaged and adapting to changing situations in fragile states, but also for strengthening local and national governance structures.

Engaging in fragile states inevitably brings along a multitude of opportunities and challenges when it comes to adopting strategies to achieve health objectives, while balancing the need not to undermine and contribute to the overall goal of mitigating fragility [10, 49]. In situations of protracted crises, uncoordinated actors with different priorities (short-term aid versus longer-term development approaches, peacebuilding agenda versus development agenda) specific sectoral interventions versus multi-sectoral and comprehensive interventions) and a persistent dependency on donor-driven initiatives with a weak governmental capacity to carry out basic functions can lead to a fragmentation of the system. This often hampers the recovery process and can easily spawn renewed conflict or perpetual fragility [50]. The results from the two case studies showed both entry points and challenges or even limitations for an individual NGO working towards mitigating fragility through a sectoral programme such as health. The multitude of exogenous factors driving fragility in both countries, linked to a different interaction with and the practices of international corporations or foreign governments within a larger geopolitical, historical, social and economic context, cannot be addressed by aid and development programmes alone [51]. Nevertheless, in post-crisis periods (e.g. after the 2010 earthquake in Haiti), opportunities arise to change the working modalities and to strengthen intersectoral collaboration and partnerships. Coherent and participatory approaches can be pursued to achieve greater ownership and equity, while capitalising on local needs, lessons learnt from the past and fostering innovation to strengthen local and national health systems, governmental accountability and legitimacy to protect their citizens [9, 50].

There are several issues from the case studies reported here that need particular attention in designing and implementing future health programmes in fragile and conflict-affected contexts. Indeed, fragile contexts are marked by rapidly changing (conflict) dynamics and vulnerabilities [52]. The transition between relief and development aid and vice versa brings together development and humanitarian actors to collaborate more closely in the same countries (e.g. common funding mechanisms, joint approaches, programmes and preparedness) [52]. Nevertheless, there are long-standing challenges and debates on-going with regards to linking relief, rehabilitation and development approaches between analysts and practitioners [10, 53]. Key issues are, amongst others, finding commonality and alignment in objectives, principles, approaches and resource allocation to identified problems of aid and development [16]. This is even more so when adding peace- and state-building approaches and objectives into the mix. Strengthening local partners who have a wide reach, such as the SSRC in South Sudan with its branches and volunteers, proved to be a critical factor that ensured a smooth transition from development aid to humanitarian activities after critical outbreaks of violence. The SRC’s long-standing partnership with the SSRC, its earlier capacity-building efforts and the long-term presence of an SRC delegate in the country had laid a foundation for cooperation before the humanitarian crisis arose, also with the signing of a framework agreement for partnership, coordination and cooperation by the Movement’s organisations (International Federation of Red Cross and Red Crescent and International Committee of the Red Cross). However, continuous efforts are necessary to improve functioning among the Movement partners. Transitions back to humanitarian activities need to be accompanied by a long-term commitment and an alignment with stakeholders across the humanitarian and development sectors to ensure, amongst other factors, continuing access to health services [54, 55].

### Recommendations

Five specific recommendations emerge from this study for better designing and implementing community-based health programmes in fragile contexts as well as for improved overall strategies and policies. First, systematic and regular training for both international staff and implementing partners on emergency response, and transition to development and preparedness for different phases, should be complemented by conflict-sensitive programme management training. Awareness of the overall fragility context (including actors, key issues and dynamics) needs to be raised, and a more thorough understanding of the programme’s interactions with the setting should be developed. This will allow project stakeholders to make informed decisions and take actions based on this newly developed and improved understanding.

Second, it is crucial to anchor programmes within already existing and established community structures and involve, as much as possible, state structures from the local up to the national levels. This will help in local capacity-building and ensure the handover of responsibility and a sustainable exit strategy and may, furthermore, help smooth the transition between development and humanitarian activities and vice versa.

Third, the issue of the sustainability of interventions has been raised at the beginning of project design stages with project partners, stakeholders and beneficiaries. Engaging in community dialogues about the intervention may not only enhance a community’s capacity to integrate interventions into existing practices, but also to address underlying elements, such as socio-cultural/community context, or the organisational settings that can support the sustainability of interventions [56]. Programmes should fit with existing community resources and should involve state authorities from the local up to the national levels to safeguard proper hand-over strategies that, moreover, promote state-led development.

Fourth, regular monitoring of the often fast-changing fragility and conflict context along with the development of a good network with local stakeholders can provide crucial information for better and readily adapted project implementation; this will help ensure best possible preparedness for any changes that are not within the control of the project stakeholders.

Fifth, advocacy for the establishment and strengthening of adequate coordination mechanisms has to be increased to avoid the creation of inequalities on the ground, which can lead to tensions and further deterioration of social cohesion. Moreover, exchange across the various aid actors (relief, development, and peace- and state-building actors) is vital for overall more coherent approaches and policies where aid organisations interfering in a context become mutually reinforcing instead of mutually weakening.

### Limitations

There are several limitations to this scoping study. First, the retrospective nature of the data collected and the length of the period covered (2008–2013 in South Sudan) may have resulted in recall bias. Second, the violent outbreaks in South Sudan shortly before the fact-finding mission may have influenced responses and limited data collection in Mayendit county. As in the case of South Sudan, only primarily programme implementers, donors or employees from SRC were interviewed, and hence the analysis does not include any beneficiary perception; consequently, there may be a bias of the study participants towards the interventions they conducted. Third, due to time constraints, only a limited number of interviews and FGDs were carried out in both countries, and the findings may thus not be representative of the perceptions of all stakeholders involved.

## Conclusions

The aim of the two case studies was to provide the SRC with an improved understanding of critical issues driving fragility in both Haiti and South Sudan and to assess whether a sectoral health programme can ultimately – beyond reaching its health targets – contribute to mitigating state fragility in the longer term. The study of the SRC’s health programmes in South Sudan and Haiti suggests that opportunities and entry points for mitigating some of the identified drivers exist, albeit to varying degrees. Challenges abound, and further investigation and reflection on some of the insights are required to get a better grasp on how far and how best to consolidate immediate health objectives with the state- and peacebuilding goals. Community-based health programmes should consider possible ways of how to promote stability and improved state–society relations. It is critical to improve awareness among health workers about their programmes’ impact on the overall context so that informed decisions can be made. Cross-sectoral collaboration needs to be strengthened, as does coordination among relief, development and peacebuilding actors who often work simultaneously in the same contexts. Ultimately, sustained engagements and strong partnerships with both key stakeholders in the fragile settings and with different international actors are indispensable to support a sustainable transition out of fragility.

## Abbreviations

CASEC: ‘Conseil d’Administration de la Section Communale’
DPC: ‘Direction de la Protection Civile’
EIC: ‘Équipes d’Intervention Communautaires’
FGD: focus group discussion
MoH: Ministry of Health
NGO: non-governmental organisations
SDC: Swiss Agency for Development and Cooperation
SRC: Swiss Red Cross
SSRC: South Sudan Red Cross
UCS: ‘Unités Communales de Santé’
WASH: water, sanitation and hygiene
